# Pre-Sleep Protein Ingestion to Improve the Skeletal Muscle Adaptive Response to Exercise Training

**DOI:** 10.3390/nu8120763

**Published:** 2016-11-28

**Authors:** Jorn Trommelen, Luc J. C. van Loon

**Affiliations:** NUTRIM School of Nutrition and Translational Research in Metabolism, Maastricht University Medical Centre+, P.O. Box 616, Maastricht 6200 MD, The Netherlands; jorn.trommelen@maastrichtuniversity.nl

**Keywords:** sleep, recovery, exercise, hypertrophy, casein

## Abstract

Protein ingestion following resistance-type exercise stimulates muscle protein synthesis rates, and enhances the skeletal muscle adaptive response to prolonged resistance-type exercise training. As the adaptive response to a single bout of resistance exercise extends well beyond the first couple of hours of post-exercise recovery, recent studies have begun to investigate the impact of the timing and distribution of protein ingestion during more prolonged recovery periods. Recent work has shown that overnight muscle protein synthesis rates are restricted by the level of amino acid availability. Protein ingested prior to sleep is effectively digested and absorbed, and thereby stimulates muscle protein synthesis rates during overnight recovery. When applied during a prolonged period of resistance-type exercise training, protein supplementation prior to sleep can further augment gains in muscle mass and strength. Recent studies investigating the impact of pre-sleep protein ingestion suggest that at least 40 g of protein is required to display a robust increase in muscle protein synthesis rates throughout overnight sleep. Furthermore, prior exercise allows more of the pre-sleep protein-derived amino acids to be utilized for de novo muscle protein synthesis during sleep. In short, pre-sleep protein ingestion represents an effective dietary strategy to improve overnight muscle protein synthesis, thereby improving the skeletal muscle adaptive response to exercise training.

## 1. Introduction

A single session of exercise stimulates muscle protein synthesis rates, and to a lesser extent, muscle protein breakdown rates [1,2]. However, the muscle protein net balance will remain negative in the absence of food intake [2]. Protein ingestion stimulates muscle protein synthesis and inhibits muscle protein breakdown rates, resulting in net muscle protein accretion during the acute stages of post-exercise recovery [3]. Therefore, post-exercise protein ingestion is widely applied as a strategy to augment post-exercise muscle protein synthesis rates and, as such, to facilitate the skeletal muscle adaptive response to exercise training. Various factors have been identified which can modulate the post-exercise muscle protein synthetic response to exercise including the amount [4,5], type [6,7], timing [8], and distribution [9] of protein ingestion.

Only few studies have investigated the dose-response relationship between protein ingestion and post-exercise muscle protein synthesis rates in young [4,5] and older adults [10,11,12]. Ingestion of 20 g egg or whey protein has been shown sufficient to maximize muscle protein synthesis rates during recovery from lower-body resistance-type exercise in young males [4,5]. More recent evidence indicates that this dose-response relationship may depend on the amount of muscle tissue that was recruited during exercise, with the ingestion of 40 g protein further increasing muscle protein synthesis rates during recovery from whole-body resistance-type exercise [13].

A large variety of dietary protein sources have been shown to stimulate post-exercise muscle protein synthesis rates, including egg protein [4], whey and casein protein [14], milk and beef protein [15], and soy protein [6]. However, dietary protein sources can differ in their capacity to stimulate muscle protein synthesis rates, which appears to be largely dependent on differences in protein digestion and absorption kinetics [14,16] and amino acid composition [6,17], with the leucine content being of particular relevance [18,19].

Besides the amount and type of ingested protein, the timing and distribution of protein ingestion throughout the day can modulate post-exercise muscle protein synthesis rates. An even distribution of total protein intake over the three main meals stimulates 24 h muscle protein synthesis rates more effectively than an unbalanced distribution in which the majority (>60%) of total daily protein intake is consumed at the evening meal [20]. During 12 h of post-exercise recovery, an intermediate pattern of protein ingestion (20 g every 3 h) seems to increase muscle protein synthesis rates to a greater extent than the same amount of protein provided in less frequent but larger amounts (40 g every 6 h), or in more frequent, smaller amounts (10 g every 6 h) [9]. Therefore, an effective pattern of daily protein intake distribution to support muscle protein synthesis is to provide at least 20 g of protein with each main meal with no more than 4–5 h between meals.

As overnight sleep is typically the longest post-absorptive period during the day, we have recently introduced the concept of protein ingestion prior to sleep as a means to augment post-exercise overnight muscle protein synthesis. The aim of this review is to discuss the current state of evidence regarding the efficacy of pre-sleep protein ingestion to stimulate overnight muscle reconditioning.

## 2. Overnight Protein Metabolism

In general, most studies assess the effects of food intake on the muscle protein synthetic response to exercise performed in an overnight fasted state. Such post-absorptive conditions differ from normal everyday practice in which recreational sports activities are often performed in the late afternoon or evening after a full day of habitual physical activity and food intake. Therefore, we evaluated the impact of exercise performed in a fed state in the evening and the efficacy of protein ingestion immediately after exercise on muscle protein synthesis during prolonged overnight recovery [21]. The ingestion of 20–25 g of protein during exercise increased muscle protein synthesis rates during exercise, but we observed no increase in muscle protein synthesis rates during the prolonged overnight recovery period. Muscle protein synthesis rates during overnight sleep were unexpectedly low, with values being even lower than those typically observed in the in the morning following an overnight fast. Thus, a day of habitual food intake and the ingestion of 20–25 g of protein during and/or immediately after an exercise bout performed in the evening does not suffice to augment overnight muscle protein reconditioning.

## 3. Does the Gut Function at Night?

As overnight muscle protein synthesis rates are surprisingly low [21], we questioned whether they are limited by overnight plasma amino acid availability. Therefore, we hypothesized that protein provision during sleep increases overnight plasma amino acid availability and stimulates overnight muscle protein synthesis rates. As human intestinal motility follows a circadian rhythm with reduced activity during the night [22], we first assessed whether dietary protein provision during sleep leads to proper dietary protein digestion and amino acid absorption. In a proof-of-principle study, we first administrated specifically produced intrinsically l-[1-^13^C]-phenylalanine-labeled casein protein via a nasogastric tube while subjects were asleep and assessed the subsequent protein digestion and absorption kinetics [23]. We observed that administration of 40 g casein via a nasogastric tube during overnight sleep is followed by proper dietary protein digestion and absorption kinetics, thereby increasing overnight plasma amino acid availability and increasing muscle protein synthesis rates. Clearly, these data demonstrated that the gut functions properly at night and that protein provided during sleep strongly increases overnight muscle protein synthesis rates.

## 4. Pre-Sleep Protein Feeding as a Strategy to Increase Overnight Muscle Protein Synthesis

**Figure 1:**
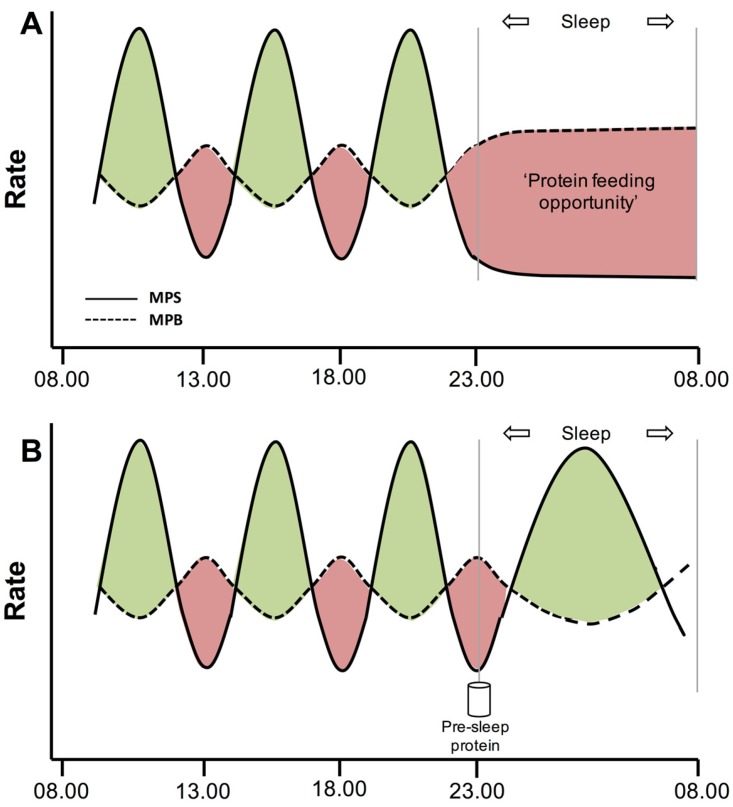
Schematic representation of the process of muscle protein synthesis (MPS) and muscle protein breakdown (MPB) throughout the day. Protein ingestion stimulates MPS rates and allows for net muscle protein accretion (green areas). During post-absorptive conditions, MPB rates exceed MPS rates, resulting in a net loss of muscle protein (red areas). Overnight sleep is the longest post-absorptive period of the day (**A**). Pre-sleep protein ingestion stimulates overnight muscle protein synthesis rates (**B**), thereby improving muscle reconditioning during overnight sleep.

Our observation that protein administered during sleep is effectively digested and absorbed provided proof-of-principle that the gut functions properly during sleep [23]. However, nasogastric tube feeding does not represent a feasible feeding strategy for athletes. Therefore, our next step was to assess if protein ingestion prior to sleep would represent an effective dietary strategy to increase muscle protein synthesis rates during overnight post-exercise recovery [24]. Therefore, we studied recreational athletes during overnight recovery from a single bout of resistance-type exercise performed in the evening after a full day of dietary standardization. Immediately after exercise, all athletes ingested a recovery drink containing 20 g protein to maximize muscle protein synthesis rates during the acute stages of post-exercise recovery [4,24]. As explained above, this prescribed recovery strategy does not suffice to maintain elevated muscle protein synthesis rates during more prolonged overnight sleep [21]. Therefore, we provided subjects with either 40 g casein protein or a placebo drink immediately prior to sleep. In line with intragastric protein administration during sleep [23], the bolus of protein ingested prior to sleep was properly digested and absorbed throughout overnight sleep. The greater plasma amino acid availability following pre-sleep protein ingestion improved the overnight whole-body protein balance, allowing the net protein balance to become positive. In line, muscle protein synthesis rates were approximately 22% higher during overnight recovery when protein was ingested prior to sleep when compared to the placebo treatment. From these data we concluded that pre-sleep protein ingestion represents an effective dietary strategy to further augment the skeletal muscle adaptive response to resistance-type exercise training (Figure 1).

To test this hypothesis, we assessed the impact of pre-sleep protein feeding to facilitate the skeletal muscle adaptive response to prolonged resistance-type exercise training [25]. Specifically, we selected healthy young men to participate in a 12-week resistance-type exercise training program (three exercise sessions per week) during which they ingested either 27.5 g of protein prior to sleep, or a non-caloric placebo. Muscle mass and strength increased to a greater extent in the group that ingested protein prior to sleep. These results indicate that protein supplementation prior to sleep represents an effective dietary strategy to augment the gains in muscle mass and strength during resistance-type exercise training. It remains to be established what dose and type of pre-sleep protein should be used to further optimize overnight muscle protein synthesis rates and, as such, can support greater gains in muscle mass and strength.

It should be noted that the ingestion of the pre-sleep protein supplement in both our acute and long-term studies was compared with a non-protein placebo, and not compared with protein supplementation provided at other time points. Therefore, we can only speculate on the surplus benefits of pre-sleep protein provision when compared to other time points. It can be speculated that the greater gains in muscle mass and strength are, at least partly, attributed to the pre-sleep timing of the protein supplement, as the vast majority of studies in which protein has been supplemented immediately before and/or after exercise do not show an increase in muscle mass gains when compared to a placebo [26]. However, it has been suggested that protein supplementation increases muscle mass gains mainly as a function of increased total protein intake, rather than the specific timing of a protein supplement [27,28]. As a meta-analysis was required to demonstrate that additional protein intake augments training-induced muscle hypertrophy [26], it seems unlikely that a possible positive effect of protein timing (i.e., protein supplementation at a time point compared to protein supplementation at different time point) on muscle mass gains can be detected in a longitudinal study. While it is currently unclear whether pre-sleep protein ingestion is superior to protein ingestion at a different time point, we propose that a more relevant question is whether pre-sleep protein ingestion is additive to protein intake earlier in the day. We suggest that athletes should aim to ingest sufficient protein intake at every meal to maximize muscle protein synthesis until the next meal. We have recently shown that the ingestion of large amounts of protein in the early post-exercise recovery phase does not compromise the muscle protein synthetic response to protein ingestion at a later stage [29]. This suggests that every meal moment represents a unique opportunity to stimulate muscle protein synthesis and that the muscle protein synthetic response to each meal may be additive. In addition, we have recently shown that athletes typically consume well above 1.2 g protein/kg/day, with the majority of protein consumed during the three main meals, and only a small amount of protein eaten as an evening snack (~7 g) [30]. As such, additional pre-sleep protein ingestion represents a practical strategy to increase the total daily protein intake, add another meal moment, and increase the overnight muscle protein synthesis rates; this effect is likely additive to muscle protein synthesis rates observed throughout the day.

## 5. Pre-Sleep Protein Feeding Characteristics

While we have identified the overnight sleeping period as a new window of opportunity to augment post-exercise training adaptations, it remains to be established how we can maximize the impact of pre-sleep protein feeding on overnight muscle protein synthesis rates. Previously we have shown that the ingestion of 40 g protein prior to sleep stimulates overnight muscle protein rates [24], which is considerably more than the 20 g of protein that is supposed to maximize muscle protein synthesis rates during the first few hours of post-exercise recovery [4,5]. Therefore, we questioned if a more moderate amount of protein would suffice to augment overnight muscle protein synthesis rates. To address this issue, we performed a follow-up study similar in design to our previous pre-sleep protein work, with the main difference that we provided 30 g of highly enriched intrinsically labeled protein prior to sleep, with or without an additional 2 g of free leucine. In contrast to our previous findings with 40 g protein, the ingestion of 30 g protein prior to sleep did not significantly increase overnight muscle protein synthesis rates (preliminary observations). This suggests that a pre-sleep protein dose-response relationship exists, which differs from the immediate post-exercise recovery period during which the ingestion of merely 20 g protein seems to maximize post-exercise muscle protein synthesis rates in young adults.

The ingestion of highly enriched, intrinsically l-[1-^13^C]-phenylalanine-labeled protein allowed us to also directly assess the metabolic fate of the pre-sleep dietary protein-derived amino acids. Pre-sleep protein-derived l-[1-^13^C]-phenylalanine was incorporated in de novo muscle protein as evidenced by the increase in muscle protein–bound l-[1-^13^C]-phenylalanine following overnight recovery, demonstrating that the pre-sleep protein provided amino acids as precursors for de novo myofibrillar protein accretion during overnight sleep. This provides mechanistic evidence to support our observation that the ingestion of 30 g protein prior to sleep augments muscle mass during three months of resistance-type exercise training [25]. However, our data suggest that at least 40 g of pre-sleep protein is required to induce a more substantial, detectable increase in muscle protein synthesis rates when assessed acutely over a 7.5 h overnight period.

As we anticipated that 30 g of pre-sleep protein might not be sufficient to adequately increase overnight muscle protein synthesis rates, we included a third treatment in which 2 g crystalline leucine was added to the 30 g bolus of protein. The addition of supplemental free leucine to a suboptimal amount of protein has been shown to enhance post-exercise muscle protein synthesis rates [18,19,31,32]. Despite these previous observations, co-ingesting free leucine with 30 g of casein prior to sleep did not augment the overnight muscle protein synthetic response. Given the extended duration of overnight sleep compared to a typical postprandial period (8 vs. 4–5 h), it is tempting to speculate that larger amounts of protein (≥40 g) are required to maximize muscle protein synthesis rates during overnight sleep.

## 6. Prior Exercise

**Figure 2:**
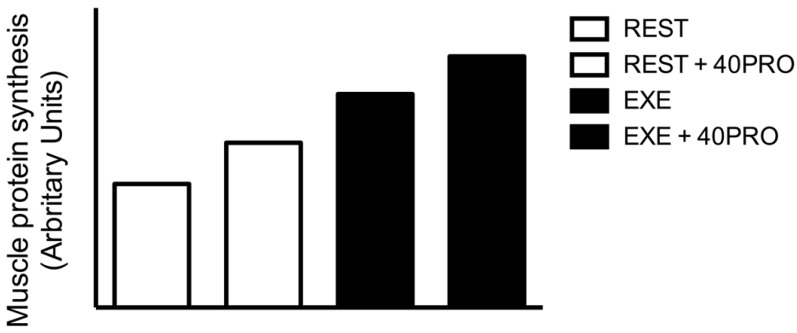
Conceptual framework of the overnight muscle protein synthetic response to 40 g of pre-sleep protein feeding at rest or following prior exercise.

It has been well established that the muscle protein synthetic response to protein ingestion is enhanced following exercise when exercise is performed in the morning following an overnight fast [12,33]. Recently, we evaluated the effect of resistance-type exercise performed in the evening on the muscle protein synthetic response to pre-sleep protein ingestion [34]. Postprandial overnight muscle protein synthesis rates were higher when exercise had been performed earlier that evening and more of the ingested protein-derived amino acids were directed towards de novo myofibrillar protein synthesis during overnight sleep. Therefore, protein ingestion prior to sleep represents an effective strategy to enhance overnight muscle reconditioning and is likely of even more relevance on exercise training days. In line, we have shown that physical activity performed in the evening increases the overnight muscle protein synthetic response to pre-sleep protein ingestion in older adults [35]. Clearly, combing pre-sleep protein ingestion with resistance-type exercise represents a more effective strategy to further enhance overnight skeletal muscle protein synthesis rates and increases the efficiency by which dietary protein is used for muscle protein accretion (Figure 2).

## 7. Type of Pre-Sleep Protein

As protein sources differ in their capacity to stimulate muscle protein synthesis, the type of protein ingested prior to sleep may modulate the overnight muscle protein synthetic response. So far, all studies assessing the efficacy of pre-sleep protein ingestion on exercise reconditioning have provided casein protein. Casein is a more slowly digestible protein source, allowing a more moderate but prolonged rise in plasma amino acid concentrations [17]. Given the extended nature of overnight sleep, it could be speculated that such a more sustained postprandial aminoacidemia during overnight sleep is preferred as it will provide precursors to support muscle protein synthesis rates throughout the entire night. In contrast, whey protein is a more rapidly digestible protein, resulting in a pronounced but transient rise in plasma amino acid concentrations [17]. Ingestion of a single bolus of whey protein has been shown to stimulate muscle protein synthesis rates to a greater degree than casein protein when assessed over periods up to 6 h [6,17,36]. This has been attributed to the more rapid protein digestion and amino acid absorption kinetics as well as the higher leucine content in whey versus casein protein, resulting in a more rapid rise in postprandial plasma leucine concentrations [37]. It remains to be established if whey is superior to casein protein when ingested prior to sleep and muscle protein synthesis rates are assessed over a more prolonged overnight period of 7.5 h. The plasma levels of leucine do not seem to be the only factor in this regard, as we recently did not observe any differences in overnight muscle protein synthesis rates following the ingestion of 30 g casein with or without 2 g crystalline leucine (preliminary observations). Snijders et al. [25] provided a casein protein supplement that consisted of 50% micellar casein and 50% casein hydrolysate. When casein protein is hydrolyzed, its digestion and absorption properties resemble a more rapid digestible protein [38]. Therefore, pre-sleep ingestion of a mixture of a slow and more rapidly digestible protein source appears to be effective to augment muscle mass and strength gains during a prolonged resistance-type exercise program. We speculate that a variety of high-quality animal-based protein sources can augment overnight muscle protein synthesis rates when provided in sufficient amounts (≥40 g; Table 1), with relatively minor differences in efficacy between sources.

## 8. Applications

Overnight sleep has emerged as a novel window of opportunity to modulate muscle protein metabolism. Pre-sleep protein ingestion represents an effective dietary strategy to stimulate both the acute and long-term skeletal muscle adaptive response to resistance-type exercise training [24,25]. There are numerous other potential applications of protein ingestion prior to sleep. Protein ingestion prior to sleep may also enhance exercise training adaptations to other exercise modalities. However, research on the impact of protein supplementation on other modes of exercise such as concurrent training [39] or endurance-type exercise training [40] is surprisingly scarce. While protein ingested immediately after endurance-type exercise does not appear to further augment mitochondrial protein synthesis rates [40], amino acid administration at rest stimulates mitochondrial protein synthesis rates [41]. It remains to be established if pre-sleep protein can augment the adaptive response to endurance-type exercise training with greater increases in skeletal muscle oxidative capacity, vascular density and/or endurance performance capacity.

Protein administration during sleep has been shown to stimulate overnight muscle protein synthesis rates in older adults [23]. Consequently, pre-sleep protein feeding may also represent an effective interventional strategy to support muscle mass maintenance in the older population or possibly even in patients in more clinically compromised conditions characterized by accelerated muscle loss such as acute sickness, systematic inflammation, and muscle disuse [42,43].

## 9. Conclusions

Muscle protein synthesis rates are particularly low during sleep, even when 20 g protein is ingested immediately after exercise performed in the evening. Protein ingested immediately prior to sleep is effectively digested and absorbed, thereby increasing amino acid availability during overnight sleep. Greater amino acid availability during sleep stimulates muscle protein synthesis rates and improves whole-body protein net balance during overnight recovery. At least 40 g of dietary protein should be ingested prior to sleep to elicit a robust stimulation of muscle protein synthesis rates throughout the night. Resistance-type exercise performed during the day augments the overnight muscle protein synthetic response to pre-sleep protein ingestion and allows more of the protein-derived amino acids to be used as precursors for de novo muscle protein synthesis. When applied during prolonged resistance-type exercise, pre-sleep protein supplementation can be used effectively to further increase gains in muscle mass and strength.

## Figures and Tables

**Table 1:** Quantity of protein sources to provide 40 g pre-sleep protein.

Food Item	Quantity
Cooked eggs	7 eggs
Low fat milk	5 cups (1025 mL)
Low fat yogurt	5 cups (1176 mL)
Chicken breast	2 breasts (176 g)
Steak	2 steaks (168 g)
Protein concentrate in water	3 scoops (60 g)
Protein concentrate in low-fat milk	2 scoops in 300 mL

## References

[1] Biolo G., Maggi S.P., Williams B.D., Tipton K.D., Wolfe R.R. (1995). Increased rates of muscle protein turnover and amino acid transport after resistance exercise in humans. Am. J. Physiol..

[2] Phillips S.M., Tipton K.D., Aarsland A., Wolf S.E., Wolfe R.R. (1997). Mixed muscle protein synthesis and breakdown after resistance exercise in humans. Am. J. Physiol..

[3] Tipton K.D., Ferrando A.A., Phillips S.M., Doyle D.J., Wolfe R.R. (1999). Postexercise net protein synthesis in human muscle from orally administered amino acids. Am. J. Physiol..

[4] Moore D.R., Robinson M.J., Fry J.L., Tang J.E., Glover E.I., Wilkinson S.B., Prior T., Tarnopolsky M.A., Phillips S.M. (2009). Ingested protein dose response of muscle and albumin protein synthesis after resistance exercise in young men. Am. J. Clin. Nutr..

[5] Witard O.C., Jackman S.R., Breen L., Smith K., Selby A., Tipton K.D. (2013). Myofibrillar muscle protein synthesis rates subsequent to a meal in response to increasing doses of whey protein at rest and after resistance exercise. Am. J. Clin. Nutr..

[6] Tang J.E., Moore D.R., Kujbida G.W., Tarnopolsky M.A., Phillips S.M. (2009). Ingestion of whey hydrolysate, casein, or soy protein isolate: Effects on mixed muscle protein synthesis at rest and following resistance exercise in young men. J. Appl. Physiol..

[7] Wilkinson S.B., Tarnopolsky M.A., Macdonald M.J., Macdonald J.R., Armstrong D., Phillips S.M. (2007). Consumption of fluid skim milk promotes greater muscle protein accretion after resistance exercise than does consumption of an isonitrogenous and isoenergetic soy-protein beverage. Am. J. Clin. Nutr..

[8] Levenhagen D.K., Gresham J.D., Carlson M.G., Maron D.J., Borel M.J., Flakoll P.J. (2001). Postexercise nutrient intake timing in humans is critical to recovery of leg glucose and protein homeostasis. Am. J. Physiol. Endocrinol. Metab..

[9] Areta J.L., Burke L.M., Ross M.L., Camera D.M., West D.W.D., Broad E.M., Jeacocke N.A., Moore D.R., Stellingwerff T., Phillips S.M. (2013). Timing and distribution of protein ingestion during prolonged recovery from resistance exercise alters myofibrillar protein synthesis. J. Physiol..

[10] Yang Y., Breen L., Burd N.A., Hector A.J., Churchward-Venne T.A., Josse A.R., Tarnopolsky M.A., Phillips S.M. (2012). Resistance exercise enhances myofibrillar protein synthesis with graded intakes of whey protein in older men. Br. J. Nutr..

[11] Yang Y., Churchward-Venne T.A., Burd N.A., Breen L., Tarnopolsky M.A., Phillips S.M. (2012). Myofibrillar protein synthesis following ingestion of soy protein isolate at rest and after resistance exercise in elderly men. Nutr. Metab..

[12] Robinson M.J., Burd N.A., Breen L., Rerecich T., Yang Y., Hector A.J., Baker S.K., Phillips S.M. (2013). Dose-dependent responses of myofibrillar protein synthesis with beef ingestion are enhanced with resistance exercise in middle-aged men. Appl. Physiol. Nutr. Metab..

[13] Macnaughton L.S., Wardle S.L., Witard O.C., McGlory C., Hamilton D.L., Jeromson S., Lawrence C.E., Wallis G.A., Tipton K.D. (2016). The response of muscle protein synthesis following whole-body resistance exercise is greater following 40 g than 20 g of ingested whey protein. Physiol. Rep..

[14] Tipton K.D., Elliott T.A., Cree M.G., Wolf S.E., Sanford A.P., Wolfe R.R. (2004). Ingestion of casein and whey proteins result in muscle anabolism after resistance exercise. Med. Sci. Sports Exerc..

[15] Burd N.A., Gorissen S.H., van Vliet S., Snijders T., van Loon L.J. (2015). Differences in postprandial protein handling after beef compared with milk ingestion during postexercise recovery: A randomized controlled trial. Am. J. Clin. Nutr..

[16] West D.W.D., Burd N.A., Coffey V.G., Baker S.K., Burke L.M., Hawley J.A., Moore D.R., Stellingwerff T., Phillips S.M. (2011). Rapid aminoacidemia enhances myofibrillar protein synthesis and anabolic intramuscular signaling responses after resistance exercise. Am. J. Clin. Nutr..

[17] Pennings B., Boirie Y., Senden J.M.G., Gijsen A.P., Kuipers H., van Loon L.J.C. (2011). Whey protein stimulates postprandial muscle protein accretion more effectively than do casein and casein hydrolysate in older men. Am. J. Clin. Nutr..

[18] Churchward-Venne T.A., Breen L., Di Donato D.M., Hector A.J., Mitchell C.J., Moore D.R., Stellingwerff T., Breuille D., Offord E.A., Baker S.K. (2014). Leucine supplementation of a low-protein mixed macronutrient beverage enhances myofibrillar protein synthesis in young men: A double-blind, randomized trial. Am. J. Clin. Nutr..

[19] Wall B.T., Hamer H.M., de Lange A., Kiskini A., Groen B.B.L., Senden J.M.G., Gijsen A.P., Verdijk L.B., van Loon L.J.C. (2013). Leucine co-ingestion improves post-prandial muscle protein accretion in elderly men. Clin. Nutr..

[20] Mamerow M.M., Mettler J.A., English K.L., Casperson S.L., Arentson-Lantz E., Sheffield-Moore M., Layman D.K., Paddon-Jones D. (2014). Dietary protein distribution positively influences 24-h muscle protein synthesis in healthy adults. J. Nutr..

[21] Beelen M., Tieland M., Gijsen A.P., Vandereyt H., Kies A.K., Kuipers H., Saris W.H.M., Koopman R., van Loon L.J.C. (2008). Coingestion of carbohydrate and protein hydrolysate stimulates muscle protein synthesis during exercise in young men, with no further increase during subsequent overnight recovery. J. Nutr..

[22] Furukawa Y., Cook I.J., Panagopoulos V., McEvoy R.D., Sharp D.J., Simula M. (1994). Relationship between sleep patterns and human colonic motor patterns. Gastroenterology.

[23] Groen B.B.L., Res P.T., Pennings B., Hertle E., Senden J.M.G., Saris W.H.M., van Loon L.J.C. (2012). Intragastric protein administration stimulates overnight muscle protein synthesis in elderly men. Am. J. Physiol. Endocrinol. Metab..

[24] Res P.T., Groen B., Pennings B., Beelen M., Wallis G.A., Gijsen A.P., Senden J.M.G., van Loon L.J.C. (2012). Protein ingestion before sleep improves postexercise overnight recovery. Med. Sci. Sports Exerc..

[25] Snijders T., Res P.T., Smeets J.S.J., van Vliet S., van Kranenburg J., Maase K., Kies A.K., Verdijk L.B., van Loon L.J.C. (2015). Protein ingestion before sleep increases muscle mass and strength gains during prolonged resistance-type exercise training in healthy young men. J. Nutr..

[26] Cermak N.M., Res P.T., de Groot L.C., Saris W.H., van Loon L.J.C. (2012). Protein supplementation augments the adaptive response of skeletal muscle to resistance-type exercise training: A meta-analysis. Am. J. Clin. Nutr..

[27] Reidy P.T., Rasmussen B.B. (2016). Role of ingested amino acids and protein in the promotion of resistance exercise-induced muscle protein anabolism. J. Nutr..

[28] Schoenfeld B.J., Aragon A.A., Krieger J.W. (2013). The effect of protein timing on muscle strength and hypertrophy: A meta-analysis. J. Int. Soc. Sports Nutr..

[29] Wall B.T., Burd N.A., Franssen R., Gorissen S.H., Snijders T., Senden J.M., Gijsen A.P., van Loon L.J.C. (2016). Pre-sleep protein ingestion does not compromise the muscle protein synthetic response to protein ingested the following morning. Am. J. Physiol. Endocrinol. Metab..

[30] Gillen J.B., Trommelen J., Wardenaar F.C., Brinkmans N.Y.J., Versteegen J.J., Jonvik K.L., Kapp C., de Vries J., van den Borne J.J.G.C., Gibala M.J. (2016). Dietary protein intake and distribution patterns of well-trained dutch athletes. Int. J. Sport Nutr. Exerc. Metab..

[31] Katsanos C.S., Kobayashi H., Sheffield-Moore M., Aarsland A., Wolfe R.R. (2006). A high proportion of leucine is required for optimal stimulation of the rate of muscle protein synthesis by essential amino acids in the elderly. Am. J. Physiol. Endocrinol. Metab..

[32] Rieu I., Balage M., Sornet C., Giraudet C., Pujos E., Grizard J., Mosoni L., Dardevet D. (2006). Leucine supplementation improves muscle protein synthesis in elderly men independently of hyperaminoacidaemia. J. Physiol..

[33] Pennings B., Koopman R., Beelen M., Senden J.M.G., Saris W.H.M., van Loon L.J.C. (2011). Exercising before protein intake allows for greater use of dietary protein-derived amino acids for de novo muscle protein synthesis in both young and elderly men. Am. J. Clin. Nutr..

[34] Trommelen J., Holwerda A.M., Kouw I.W.K., Langer H., Halson S.L., Rollo I., Verdijk L.B., van Loon L.J.C. (2016). Resistance exercise augments postprandial overnight muscle protein synthesis rates. Med. Sci. Sports Exerc..

[35] Holwerda A.M., Kouw I.W. K., Trommelen J., Halson S.L., Wodzig W.K., Verdijk L.B., van Loon L.J. (2016). Physical activity performed in the evening increases the overnight muscle protein synthetic response to presleep protein ingestion in older men. J. Nutr..

[36] Burd N.A., Yang Y., Moore D.R., Tang J.E., Tarnopolsky M.A., Phillips S.M. (2012). Greater stimulation of myofibrillar protein synthesis with ingestion of whey protein isolate v. micellar casein at rest and after resistance exercise in elderly men. Br. J. Nutr..

[37] Devries M.C., Phillips S.M. (2015). Supplemental protein in support of muscle mass and health: Advantage whey. J. Food Sci..

[38] Koopman R., Crombach N., Gijsen A.P., Walrand S., Fauquant J., Kies A.K., Lemosquet S., Saris W.H.M., Boirie Y., van Loon L.J.C. (2009). Ingestion of a protein hydrolysate is accompanied by an accelerated in vivo digestion and absorption rate when compared with its intact protein. Am. J. Clin. Nutr..

[39] Perez-Schindler J., Hamilton D.L., Moore D.R., Baar K., Philp A. (2015). Nutritional strategies to support concurrent training. Eur. J. Sport Sci..

[40] Moore D.R., Camera D.M., Areta J.L., Hawley J.A. (2014). Beyond muscle hypertrophy: Why dietary protein is important for endurance athletes 1. Appl. Physiol. Nutr. Metab..

[41] Bohe J., Low A., Wolfe R.R., Rennie M.J. (2003). Human muscle protein synthesis is modulated by extracellular, not intramuscular amino acid availability: A dose-response study. J. Physiol..

[42] Kortebein P., Ferrando A., Lombeida J., Wolfe R., Evans W.J. (2007). Effect of 10 days of bed rest on skeletal muscle in healthy older adults. JAMA.

[43] Klaude M., Mori M., Tjader I., Gustafsson T., Wernerman J., Rooyackers O. (2012). Protein metabolism and gene expression in skeletal muscle of critically ill patients with sepsis. Clin. Sci..

